# Efficacy of Integrated Traditional Chinese and Western Medicine for Treating COVID-19: A Systematic Review and Meta-Analysis of RCTs

**DOI:** 10.3389/fpubh.2021.622707

**Published:** 2021-07-08

**Authors:** Bei Yin, Yi-Ming Bi, Lu Sun, Jin-Zhu Huang, Jia Zhao, Jia Yao, An-Xiang Li, Xian-Zhe Wang, Guan-Jie Fan

**Affiliations:** ^1^School of Second Clinical Medicine, Guangzhou University of Chinese Medicine, Guangzhou, China; ^2^Department of Endocrinology, The Second Affiliated Hospital of Guangzhou University of Chinese Medicine, Guangzhou, China; ^3^Department of Endocrinology, Guangdong Provincial Hospital of Chinese Medicine, Guangzhou, China

**Keywords:** integrated Chinese and Western medicine, COVID-19, efficacy, systematic review, meta-analysis

## Abstract

**Background:** Integrated Chinese and Western medicine (integrated medicine) is routinely used in the treatment of coronavirus disease 2019 (COVID-19) in China. In this study, we undertook a systematic review and meta-analysis of published randomized controlled trials (RCTs) to evaluate the efficacy of integrated medicine therapy for patients with COVID-19.

**Methods:** In this meta-analysis, we searched PubMed, Embase, Web of Science, SinoMed, China National Knowledge Infrastructure (CNKI), Chongqing VIP (CQVIP), and Wanfang databases from inception to April 12, 2021, to identify RCTs of integrated medicine in the treatment of COVID-19. The quality of RCTs was assessed by the Cochrane risk of bias tool. RevMan v5.3 and Stata software packages were used for statistical analysis.

**Results:** Nineteen RCTs involving 1,853 patients met our inclusion criteria. Compared with patients treated by conventional Western medicine (CWM), patients treated by integrated medicine have a higher overall effective rate [*RR* = 1.17, 95% CI: (1.10, 1.26), *p* < 0.00001], fever disappearance rate [*RR* = 1.25, 95% CI: (1.04, 1.50), *p* = 0.02], fatigue disappearance rate [*RR* = 1.28, 95% CI: (1.00, 1.63), *p* = 0.05], and chest CT improvement rate [*RR* = 1.24, 95% CI: (1.14, 1.34), *p* < 00001]. Beneficial effects of the integrated medicine therapy were also seen in C-reactive protein (CRP) level [*WMD* = −4.14, 95% CI: (−6.38, −1.91), *p* = 0.0003] and white blood cell (WBC) count [WMD = 0.35, 95% CI: (0.11, 0.58), *p* = 0.004]. Subgroup analyses showed that, when the treatment time is <2 weeks, the effect of integrated medicine treatment is more obvious in improving the overall effective rate, clinical symptoms (fever, fatigue, and cough), the CRP level, and WBC count compared with that of the CWM treatment. For patients with severe and non-severe COVID-19, integrated medicine is more effective in improving fever and cough symptoms and WBC count than using CWM alone.

**Conclusion:** The results of the current meta-analysis suggested that the integrated medicine can improve the clinical symptoms, chest CT and infection indicators of COVID-19 patients. Even if the treatment time is <2 weeks, the effect of integrated medicine in improving symptoms is more obvious compared with the treatment of CWM. However, the results should be interpreted cautiously due to the heterogeneity among the studies.

## Introduction

Since the occurrence of the novel coronavirus pneumonia (NCP) in December 2019, the situation has increasingly become severe, and the disease continues to spread, which has had a significant impact on health and lives of people (1). Coronavirus disease 2019 (COVID-19) is a highly contagious viral pneumonia caused by severe acute respiratory syndrome coronavirus 2 (SARS-CoV-2) (2). People are usually susceptible to SARS-CoV-2, and there are different clinical manifestations (3). Mild symptoms usually include fever, dry cough, diarrhea, and fatigue. Patients with severe symptoms will rapidly develop acute respiratory distress syndrome (ARDS), multiple organ failure (MODS), and even death (3, 4). Globally, SARS-CoV-2 has infected more than 100 million people and has claimed 3.68 million lives worldwide but continues to cause effect, according to a report from the World Health Organization (as of 5:18 p.m. CEST, June 3, 2021) (5). Many COVID-19 vaccines have been developed at an unprecedented rate to prevent the occurrence of COVID-19 (5). However, apart from conventional Western medicines (CWMs), such as antiviral, antibacterial, expectorants, and bronchodilators, there is no specific drug for SARS-CoV-2, and COVID-19-targeting inhibitors are still under development (6). Given the complexity of the COVID-19, we should make more efforts to understand the pathophysiology of this new disease and look for alternative therapies that are novel, safe, and effective.

It is worth noting that the third edition of the COVID-19 diagnosis and treatment plan edited by the National Health Commission of China proposed the application of Chinese medicine (7). Traditional Chinese medicine (TCM) learns from the anti-epidemic experience accumulated in traditional medicine and may prevent the occurrence and development of diseases. Studies have shown that TCM has the characteristics of multicomponents acting on multitargets at multipathways and with broad-spectrum antiviral, anti-inflammatory activity, immunomodulatory, and organ-protective effects in the treatment of COVID-19 (8, 9). The integrated TCM and CWM (hereafter referred to as “integrated medicine”) therapy as a key component of the COVID-19 treatment regimen effectively prevented the spread of the COVID-19 epidemic in China (7).

Previously published meta-analysis found that Chinese herbal medicines or integrated medicine therapy had better effects in the treatment of COVID-19, but conclusions were limited by the relatively high heterogeneity and low accuracy of the data included (10–14). With the increase in the publications on the latest COVID-19 research, in order to test the efficacy of integrated medicine to the greatest extent, we conducted a systematic review and meta-analysis to objectively evaluate the effectiveness of integrated medicine in the treatment of COVID-19.

## Materials and Methods

### Systematic Search

We conducted a comprehensive search of seven different electronic databases, namely, PubMed, Excerpta Medica Database (Embase), Web of Science, SinoMed, China National Knowledge Infrastructure (CNKI), Chongqing VIP (CQVIP), and Wanfang Databases from inception to April 12, 2021, for randomized controlled trials (RCTs) investigating the role of integrated medicine in patients with COVID-19. We developed the search strategy with the assistance of an expert medical librarian, and the search terms were as follows: COVID-19, coronavirus disease 2019, SARS-CoV-2, novel coronavirus pneumonia, NCP, novel coronavirus, Chinese herbal medicine, traditional Chinese medicine, classical Chinese herbal formulas, Chinese herb, and medicine. We used the Medical Subject Headings database for the identification of synonyms and combined them with keywords as a search strategy (Supplementary Appendix). We also checked the references in the list of eligible publications for other related articles. No limits were set on language, publication year, or type of publication.

### Study Inclusion Criteria

The inclusion criteria were constructed in accordance with the principle of PICOS. (1) Participants: patients with COVID-19 were included in the study, and the gender, age, and nationality of the patient were not restricted. (2) Type of interventions: the treatment group was treated with integrated TCM and CWM. The dosage forms of TCM included decoction, tablet, pill, powder, granule, capsule, cream, oral liquid, plaster, and injection. The CWM treatment in both the treatment group and the control group had to be the same in terms of usage and dosage. (3) Type of controls: the control group was treated with CWM, including antiviral, antibacterial, antitussive, expectorant, and antiasthmatic drugs and symptomatic supportive treatment. (4) Outcomes: the primary outcome measure was: overall effective rate; the secondary outcome measures were fever disappearance rate, fatigue disappearance rate, cough disappearance rate, chest CT improvement rate, C-reactive protein (CRP) (mg/L), erythrocyte sedimentation rate (ESR), procalcitonin (PCT) (ng/L), white blood cell (WBC) count (10^9^/L), and lymphocyte (LY) count (10^9^/L). (5) Study design: RCTs were eligible.

Overall effective rate = (clinical recovery cases + significantly effective cases + effective cases)/total cases × 100%. According to “Evaluation standard of curative effects of traditional Chinese medicine on COVID-19” (15), the curative effect is divided into: Clinical recovery: clinical symptoms and signs of TCM disappeared or basically disappeared, and the score decreased by ≥90%; significantly effective: TCM clinical symptoms, signs improved significantly, 70% ≤ score decreased <90%; effective: TCM clinical symptoms and signs were improved, 30% ≤ score decreased <70%; invalid : TCM clinical symptoms, signs were not significantly improved, or even worse, scores decreased <30%. Score changes refer to “Evaluation standard of curative effects of traditional Chinese medicine on COVID-19” (15), including symptoms such as fever, cough, and fatigue and are scored according to the severity.

### Study Elimination Criteria

The elimination criteria were as follows: (1) duplicate studies; (2) studies in which the experimental group was subjected to other TCM therapies, such as acupuncture, moxibustion, cupping, massage, qigong, and taiji therapy, in addition to the CWM; (3) studies in which the control group was treated with a form of TCM or integrated medicine; (4) studies in which data could not be extracted.

### Data Extraction and Management

After removing duplicates, two reviewers (BY and YB) independently screened the titles and abstracts of each study in accordance with the inclusion and exclusion criteria. The full texts were subsequently obtained and evaluated by two reviewers (BY and YB) separately. Any discrepancies were resolved by consensus through discussion with the corresponding author (GF). Two reviewers (BY and YB) extracted the data from the included studies independently and double-checked the data using prepared data extraction forms, including authors, publication date, journal, title, sample size, study design, mean age, diagnostic criteria, subtypes of COVID-19, detailed information on methodology, intervention details, duration of treatment, and outcome measures.

All included pieces of literature were managed by Endnote (Version X8). When relevant details were insufficiently reported in studies, the authors were contacted by e-mail or phone if necessary.

### Quality Assessment of Included Studies

In accordance with the Cochrane Collaboration's update tool for assessing the risk of bias (16), two reviewers (BY and YB) assessed the quality of the studies independently, and disagreements were resolved by discussion or consultation with the corresponding author (GF). The evaluation of the methodological quality of each item included random sequence generation, allocation concealment, blinding of outcome assessment, blinding of participants and personnel, incomplete outcome data, selective outcome reporting, and other forms of bias.

### Statistical Analysis

Based on the Cochrane Handbook for Systematic Reviews of Interventions, the SDs of the change from baseline to post-therapy were calculated using the following formula (*R* = 0.5):

$$\begin{array}{l} SD\left(C\right)=\sqrt{SD\left(B\right)\wedge 2+\;SD\left(F\right)\wedge 2-\;\left(2*R\;*SD\left(B\right)\;*\;SD\left(F\right)\right)} \end{array}$$

where SD (B), SD (F), and SD (C) represent the SDs of the baseline, final, and change, respectively; from continuous data, we took a weighted mean difference (WMD) with 95% confidence interval (CI), while dichotomous data were expressed as relative risk (RR) with 95% CI. Statistical heterogeneity was tested by the χ^2^-based Cochran Q statistic and *I*^2^ statistic. If *I*^2^ was ≤ 50% and *p* > 0.10, we used a fixed-effects model to pool the estimations across the studies, where, *I*^2^ score >50% or *p* ≤ 0.10 indicates important heterogeneity. A random-effects statistical model was used when data showed significant heterogeneity.

As long as there is significant heterogeneity, we search for potential sources of heterogeneity. For example, if the results of a study are completely out of the range of the other studies, then we will look for possible reasons to explain the difference and conduct a sensitivity analysis to investigate the causes of heterogeneity in methodological quality. Subgroup analysis was planned to assess the impact on heterogeneity from different clinical trials where possible, including studies with treatment duration (<2 weeks and ≥2 weeks), subtypes of COVID-19 (severe type of COVID-19 and non-severe types of COVID-19), and risk bias for sequence generation (low risk for sequence generation and unclear risk for sequence generation).

Moreover, potential publication bias was assessed by Begg's tests. Results were considered as statistically significant for *p* <0.05. All statistical analyses were performed using Stata (Version 14.2, Stata Corporation, College Station, TX, United States) and RevMan (Version 5.3.0, Copenhagen: The Nordic Cochrane Centre, The Cochrane Collaboration, 2014).

## Results

### Search Results and Study Characteristics

**Figure 1:**
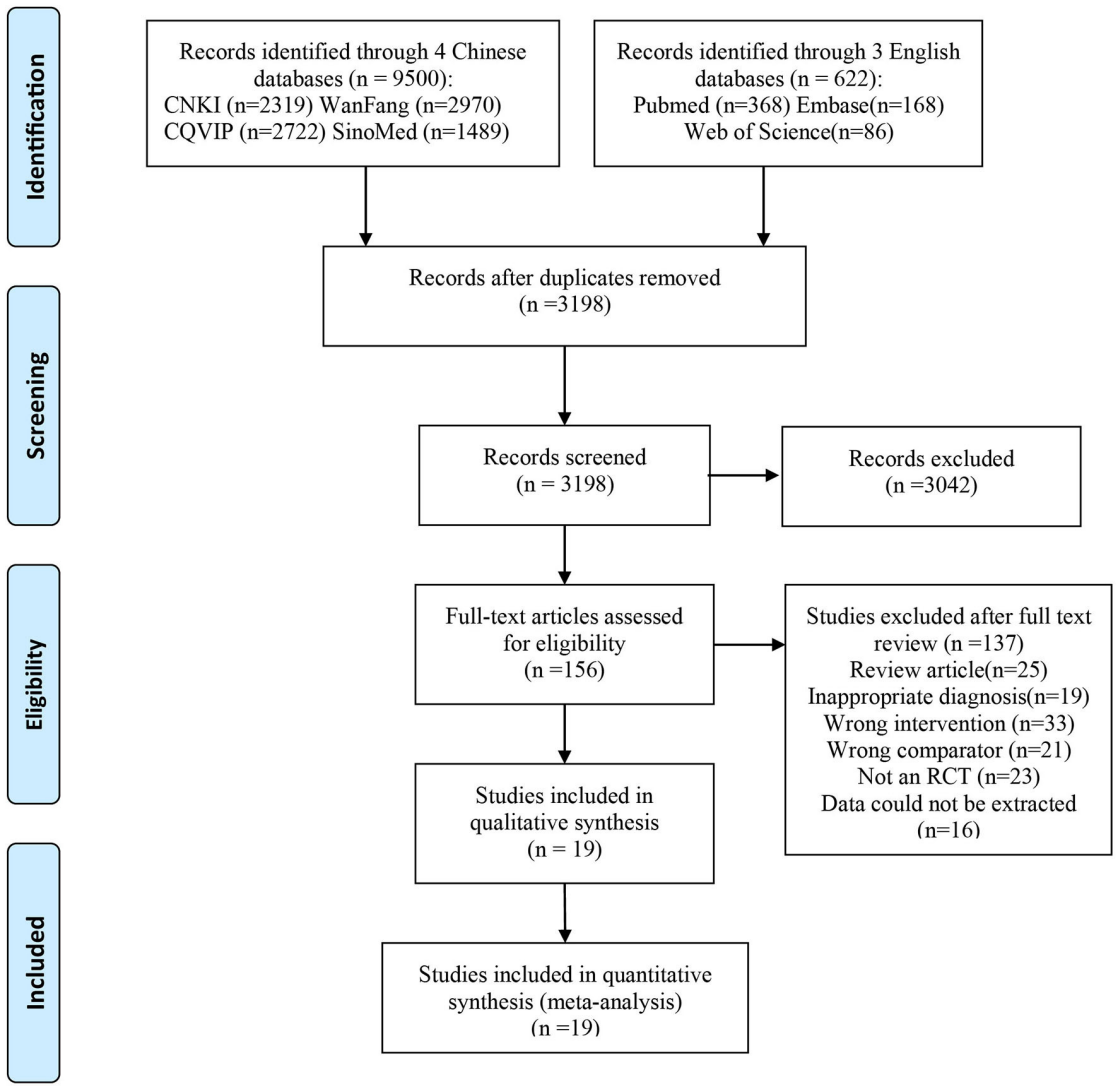
Flow diagram of study selection.

As shown in Figure 1, the search of the electronic databases and reference lists yielded a total of 10,122 potentially relevant citations, of which 6,924 were duplicates and 3,042 were excluded after screening the titles and abstracts. We assessed 153 full-text articles and included 19 RCTs in the review (17–35). Finally, a total of 19 studies met the inclusion criteria and were included for qualitative synthesis and systematic review. Baseline characteristics of the included studies are depicted in Table 1.

**Table 1:** Baseline characteristics of the included studies.

**Study author**	**Study design**	**Registration number**	**Types of COVID-19**	**Sample size**		**Intervention**	**Comparison**	**Duration (days)**	**Outcomes**
				**(I)**	**(C)**				
1. Ai et al. (18)	Single-site RCT	-	Ordinary	33	34	Pneumonia No.1 prescription, pneumonia recovery formula (100 ml, two times/day) + CWM	CWM including A, B, C, D	12	②③④
2. Ding et al. (19)	Single-site RCT	-	Mild, ordinary, severe, critical	51	49	Qingfei Touxie Fuzheng recipe (150 ml, two times/day) + CWM	CWM including A, B, C, D	10	②④⑤⑥⑦
3. Duan et al. (20)	Single-site RCT	-	Mild	82	41	Jinhua Qinggan granules (2 bag, three times/day) + CWM	CWM including A, B	5	②③④
4. Fu et al. (22)	Single-site RCT	-	Mild, Ordinary	32	33	Toujie Quwen granule (one dose, two times/day) + CWM	CWM including A, B, C, D	10	①⑤⑥⑧⑨⑩
5. Fu et al. (21)	Single-site RCT	-	Ordinary	37	36	Toujie Quwen granule (one dose, two times/day) + CWM	CWM including A, C, D	15	①⑥⑨⑩
6. Li and Zhang (25)	Single-site RCT	-	Severe	6	6	Qingfei Paidu decoction (one dose, two times/day) + CWM	CWM including A, B, C, D	6	①⑨
7. Qiu et al. (27)	Single-site RCT	-	Ordinary	25	25	Maxing Xuanfei Jiedu decoction (150 ml, three times/day) + CWM	CWM including A	10	⑤
8. Yu et al. (31)	Single-site RCT	-	Mild, Ordinary	147	148	Lianhua Qingwen granule (30 mg, three times/day) + CWM	CWM including A, B, C	7	⑤⑥⑧⑨⑩
9. Zhang et al. (32)	Single-site RCT	-	Ordinary	80	40	Honeysuckle oral liquid (50 ml, three times/day) + CWM	CWM including A, C, D	10	②③④
10. Hu et al. (17)	Multiple-site RCT	ChiCTR 2000029434	Ordinary	142	142	Lianhua Qingwen granule (4 capsules, three times/day) + CWM	CWM including A, B, D, E	14	①⑤
11. He et al. (23)	Single-site RCT	–	Mild	36	35	Buzhong Yiqi decoction (one dose, two times/day) + CWM	CWM including A	10	⑦
12. Jin et al. (24)	Multiple-site RCT	ChiCTR 2000029558	Ordinary	20	18	Compound Yin Chai granule + Qingqiao detoxification granule (15 g, four times/day) + CWM	CWM including A, B, C, D	21	②③④⑤⑥⑧⑨
13. Liu et al. (26)	Single-site RCT	-	Mild	44	44	Lianhua Qingwen capsule (1.4 g, three times/day) + pneumonia 2 concerted prescription (one dose, two times/day) + CWM	CWM including A	21	①
14. Sun et al. (28)	Single-site RCT	-	Mild, Ordinary	32	25	Lianhua Qingke granule (1 bag, two times/day) + CWM	CWM including A, C, D	14	②③④⑤
15. Wang et al. (29)	Single-site RCT	-	Ordinary	40	40	Shengmai powder + Shenling Baizhu powder (200 ml, two times/day) + CWM	CWM including A, C, D	-	①⑤⑥⑦⑧⑨⑩
16. Wang et al. (30)	Single-site RCT	-	Ordinary	70	70	Qingfei Paidu decoction (100 ml, 2 times/day) + CWM	CWM including A, B, C, D	10	⑥⑨
17. Wang et al. (33)	Single-site RCT	NCT 04251871	-	24	23	Keguan-1 (19.4g, two times/day) + CWM	CWM including A, D	14	⑤
18. Wu et al. (35)	Single-site RCT	ChiCTR 2000034795	Mild, ordinary, severe	22	20	Xuanfei Baidu decoction (200 ml, two times/day) + CWM	CWM	7	②③④
19. Xiao et al. (34)	Single-site RCT	ChiCTR 2000029601	-	58	63	Lianhua Qingwen granule (1 bag, three times/day) + CWM	CWM including A, B	14	③④

*T, treatment group; C, control group; RCT, randomized controlled trial; CWM, conventional Western medicine; A, antiviral medications; B, antimicrobial medication; C, symptomatic therapies (expectorant, antitussive drugs); D, supportive therapy (gamma globulin, methylprednisolone); E, immunosuppressant; ① Overall effective rate; ② Fever disappearance rate; ③ Fatigue disappearance rate; ④ Cough disappearance rate; ⑤ Chest CT improvement rate; ⑥ C-reactive protein (CRP) (mg/L); ⑦ Erythrocyte sedimentation rate (ESR); ⑧ Procalcitonin (PCT) (ng/L); ⑨ White blood cell count (WBC) (10^9^/L); ⑩ Lymphocyte count (LY) (10^9^/L)*.

The included studies were conducted in China between 2020 and 2021. Among them, 15 studies were published in Chinese literature and 4 in English literature. In these studies, 1,853 participants were included; the sample sizes ranged from 6 to 147, and the follow-up duration ranged from 5 to 21 days. The COVID-19 subtypes of the participants included in this study mainly include four types, such as mild, ordinary, severe, and critical, not including rehabilitation patients. The treatment groups in the included studies were treated with integrated medicine, while the control groups were treated with CWM.

### Risk of Bias

**Figure 2:**
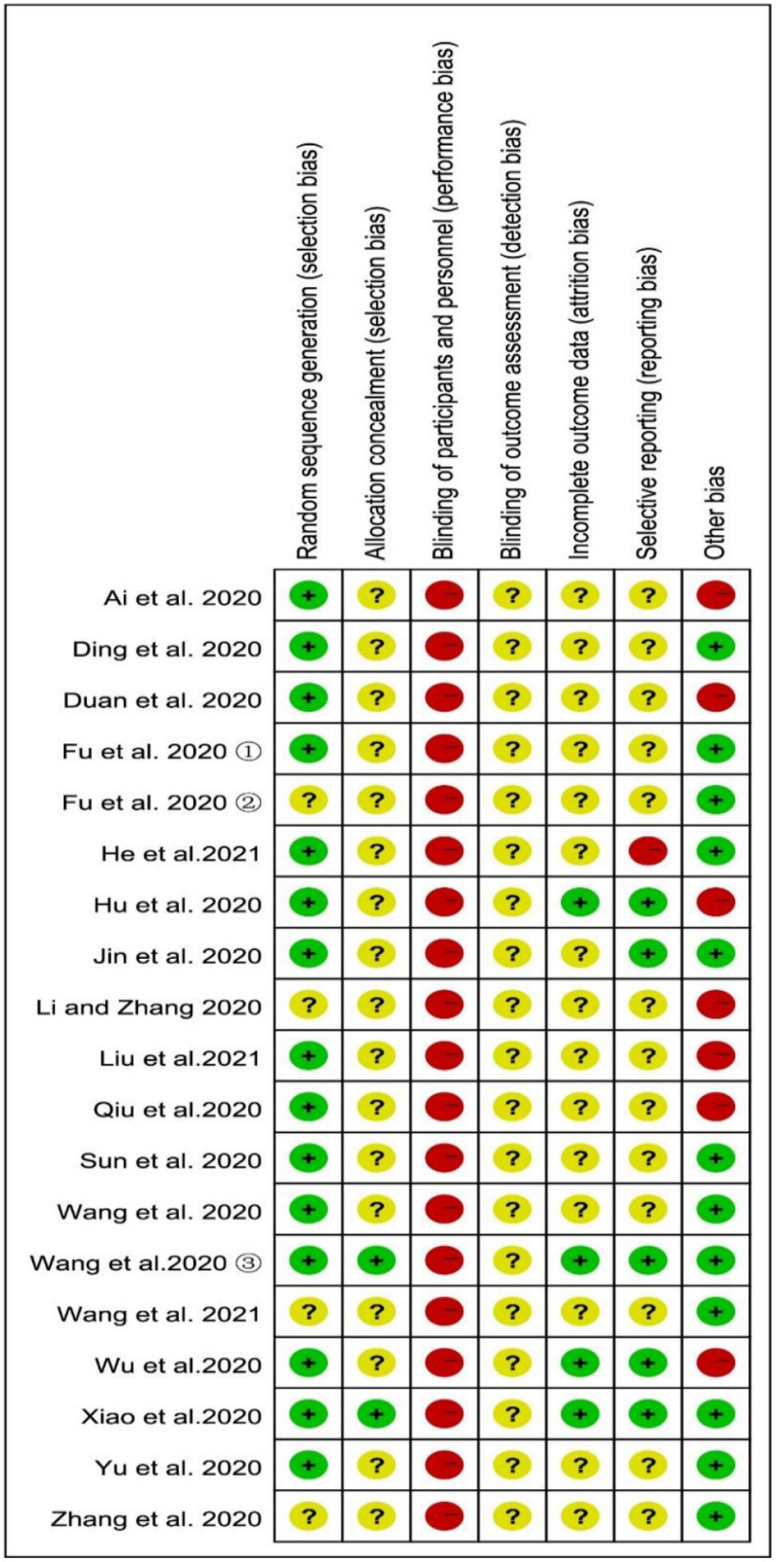
Risk of bias summary.

According to the prespecified criteria, in the 19 included studies, the participants were randomly assigned to the integrated medicine group or CWM group; only four studies (22, 25, 30, 32) did not describe the method of randomization and were categorized as unclear risk. Except for two studies (33, 34), none of the studies provided information about allocation concealment and were categorized as an unclear risk in selection bias. The most common weaknesses in the study methods were that none of the studies described blinding of outcome assessment, so they were evaluated as an unclear risk in detection bias. Furthermore, drugs were administered in different ways in the treatment and control groups in all the studies, and blinding in participants and personnel was easily broken. Therefore, all the studies were categorized as high risk in performance bias. Fifteen studies had incomplete outcome data and no follow-up, so they were classified as studies with unclear risk in attrition bias, while the remaining studies (17, 33–35) were classified as low risk because they had reported exclusions and the number of cases. Only one study (23) was classified as high risk in reporting bias, since the study did not report all the outcome indicators described in the methodological section; five studies (17, 24, 33–35) were classified as low risk because they have been clinically registered in the Chinese Clinical Trial Registry (ChiCTR) or USA National Institutes of Health Register (ClinicalTrials.gov) and had a registration number; and the remaining 13 studies were categorized as unclear risk in reporting bias since it is unclear whether an RCT is registered. The risk of other bias was considered high in the seven studies (17, 18, 20, 25–27, 35) because the drug dose of the control group is unknown, while other studies had complete data and no other bias. The summaries of the risk of bias assessment are illustrated in Figure 2.

### Outcome of Integrated Medicine for COVID-19

#### Primary Outcome Measure

##### Overall Effective Rate

**Figure 3:**
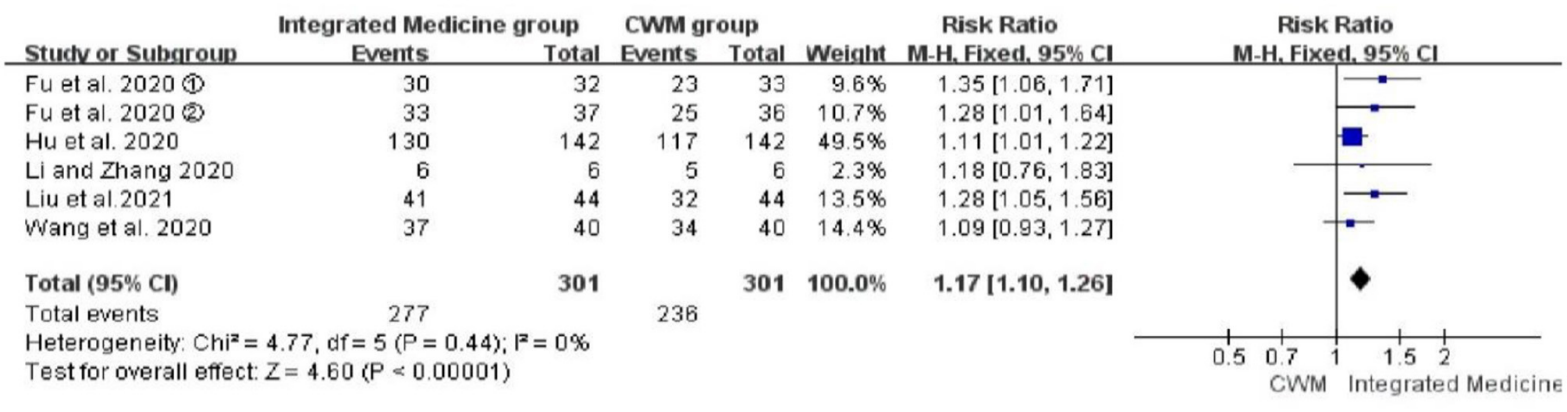
Forest plot of the overall effective rate.

Six studies evaluated the effects of integrated medicine on the overall effective rate (17, 21, 22, 25, 26, 29). There were 301 patients in the integrated medicine group and 301 in the CWM group. Integrated medicine exhibited a significant improvement on the overall effective rate [*RR* = 1.17, 95% CI: (1.10, 1.26), *p* < 0.00001] (Figure 3).

#### Secondary Outcome Measures

##### Fever, Fatigue, and Cough Disappearance Rate

**Figure 4:**
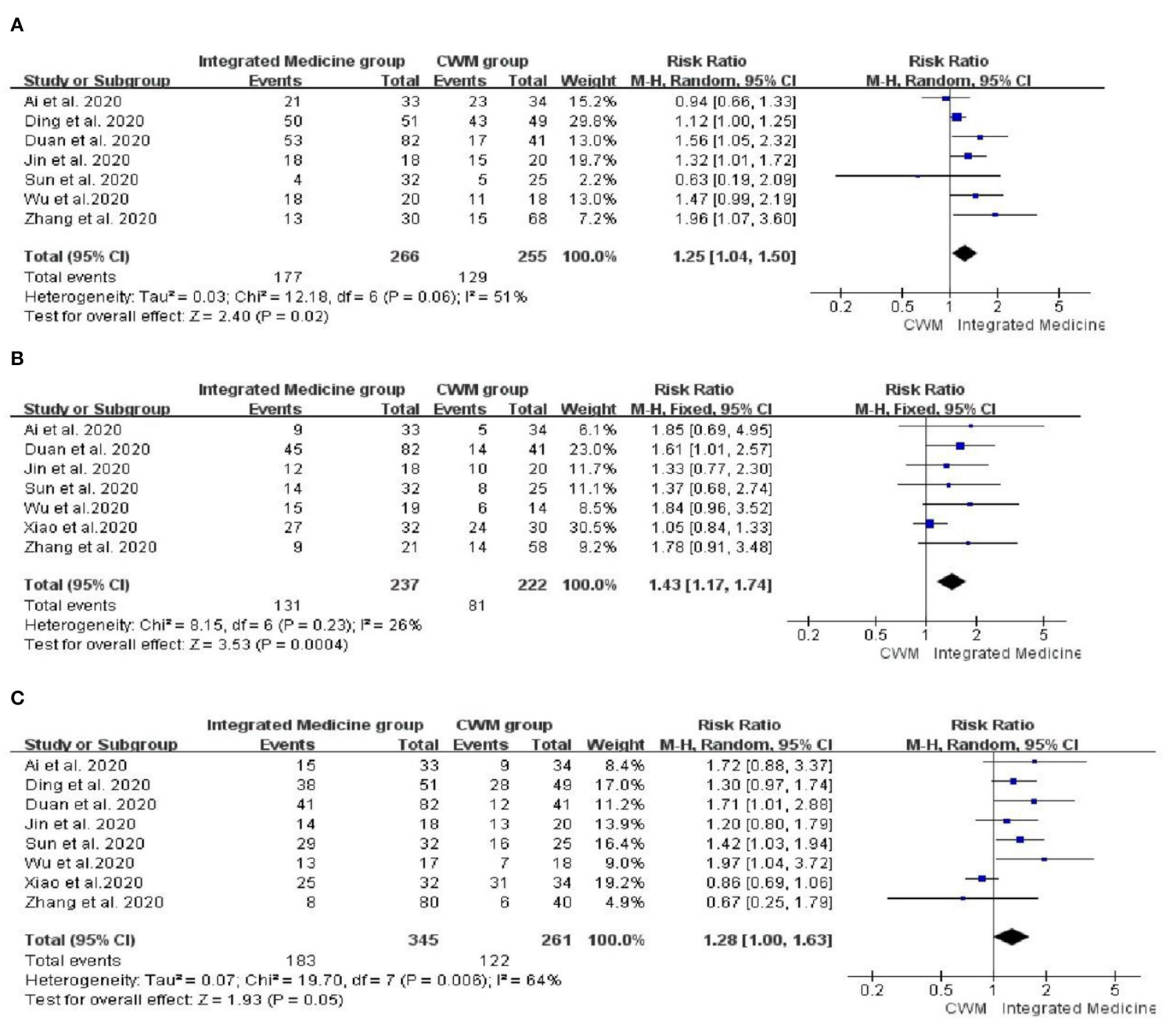
Forest plot of fever disappearance rate **(A)**, fatigue disappearance rate **(B)** and cough disappearance rate **(C)**.

Seven studies (18–20, 24, 28, 32, 35) involving 521 patients reported the fever disappearance rate after treatment. Seven studies (18, 20, 24, 28, 32, 34, 35) involving 429 patients reported the fatigue disappearance rate after treatment. Eight studies (18–20, 24, 28, 32, 34, 35) including 606 participants reported the cough disappearance rate after treatment. Compared with patients treated with CWM, patients treated with integrated medicine have a higher fever disappearance rate [*RR* = 1.25, 95% CI: (1.04, 1.50), *p* = 0.02] (Figure 4A) and fatigue disappearance rate [*RR* = 1.43, 95% CI (1.17, 1.74), *p* = 0.0004] (Figure 4B). Besides, as for the cough disappearance rate, there were no significant differences between integrated medicine and CWM [*RR* = 1.28, 95% CI: (1.00, 1.63), *p* = 0.05] (Figure 4C). The pooled analysis showed no statistical heterogeneity among the included studies of fatigue disappearance rate (*p* = 0.23, *I*^2^ = 26%). However, significant heterogeneity was observed in cough disappearance rate (*p* = 0.006, *I*^2^ = 64%) and fever disappearance rate (*p* = 0.06, *I*^2^ = 51%).

##### Chest CT Improvement Rate

**Figure 5:**
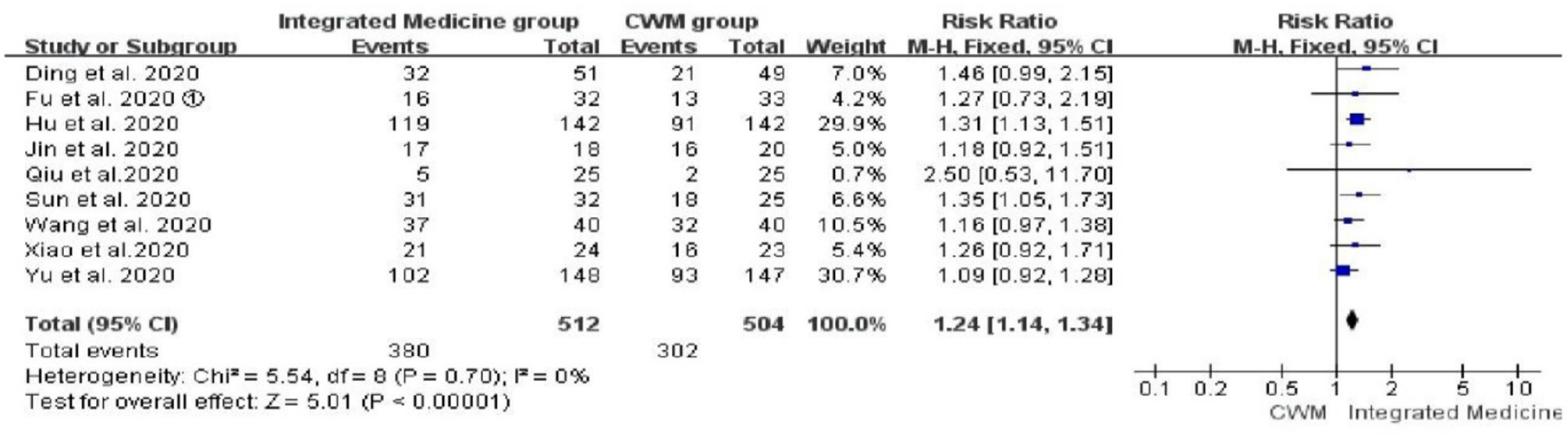
Forest plot of chest CT improvement rate.

Nine studies (17, 19, 22, 24, 27, 28, 31, 33, 34) reported the comparison of chest CT improvement rate between integrated medicine treatment and CWM treatment, and no heterogeneity was observed (*p* = 0.70, *I*^2^ = 0%). There were 512 patients in the integrated medicine group and 504 in the CWM group. Meta-analysis suggested that chest CT improvement rate is significantly improved by integrated medicine treatment [*RR* = 1.24, 95% CI: (1.14, 1.34), *p* < 00001] (Figure 5).

##### CRP

**Figure 6:**
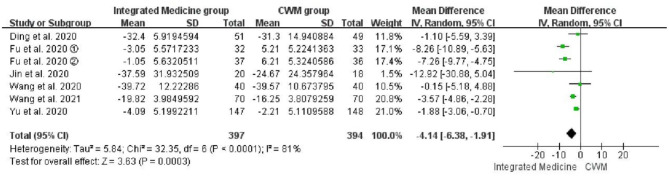
Forest plot of CRP (mg/L).

Seven studies (19, 21, 22, 24, 29–31) evaluated the therapeutic effects of integrated medicine on the CRP level. There were 397 patients in integrated medicine group and 394 in the CWM group. The meta-analysis demonstrated that the integrated medicine was superior to the CWM in improving the CRP level [*WMD* = −4.14, 95% CI: (−6.38, −1.91), *p* = 0.0003] (Figure 6). However, significant heterogeneity was observed among the included studies for CRP (*p* < 0.0001, *I*^2^ = 81%).

##### WBC

**Figure 7:**
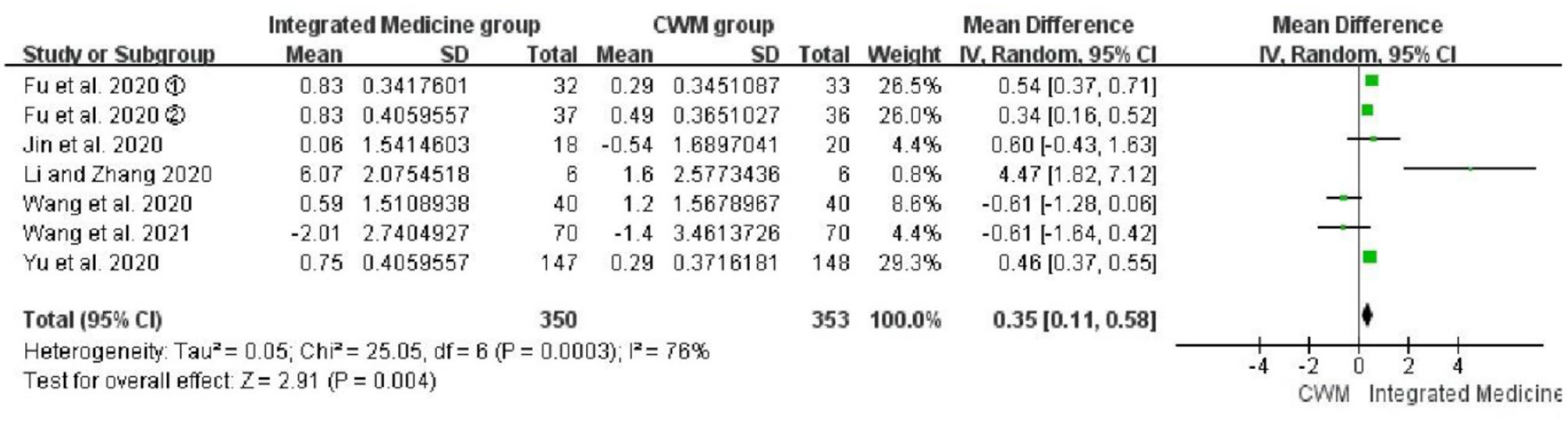
Forest plot of white blood cell (10^9^/L) count.

Seven studies (21, 22, 24, 25, 29–31) involving 703 patients were included to evaluate the efficacy of the integrated medicine on WBC count. Meta-analysis suggested that WBC count was significantly improved by integrated medicine treatment [*WMD* = 0.35, 95% CI: (0.11, 0.58), *p* = 0.004] (Figure 7), but the heterogeneity was high among the included studies for WBC (*p* = 0.0003, *I*^2^ = 76%).

##### ESR, PCT, and LY

Three studies (19, 23, 29) reported on the improvement of the ESR level, where two studies (19, 23) showed favorable effects of integrated medicine treatment for the ESR (*p* < 0.05), and the other study (29) reported no significance. Four studies (22, 24, 29, 31) evaluated the therapeutic effects of integrated medicine on the PCT level after the intervention, where two studies (22, 31) showed a positive effect toward integrated medicine treatment for the PCT (*p* < 0.05), and the other two studies (24, 29) reported no significance. Four studies (21, 22, 29, 31) reported the effect of integrated medicine on LY and showed favorable effects of integrated medicine treatment for LYs (*p* < 0.05).

### Sensitivity Analysis

Significant heterogeneity was observed among the included studies for the fever disappearance rate (*I*^2^ = 51%), cough disappearance rate (*I*^2^ = 64%), CRP (*I*^2^ = 81%), and WBC (*I*^2^ = 76%). Therefore, we conducted a sensitivity analysis to investigate the source of the heterogeneity. The study conducted by Xiao et al. did not mention the different subtypes of COVID-19 cases, and the heterogeneity may be caused by different degrees of severity of the disease in the participant. After excluding that study, the heterogeneity decreased significantly (*I*^2^ = 0%), and the results showed that the cough disappearance rate was significantly improved by integrated medicine treatment [*RR* = 1.41, 95% CI: (1.18, 1.68), *p* = 0.0001] (Supplementary Figure 1). Sensitivity analysis shows that excluding any study for each result will not change the fever disappearance rate, CRP, and WBC results, indicating that the conclusion is reliable.

### Subgroup Analysis

#### Subgroup Analysis Based on Treatment Duration

Subgroup analysis showed that the integrated medicine treatment significantly improved the overall effective rate, CRP, and WBC compared with CWM treatment, regardless of whether the treatment time exceeds 2 weeks (*p* < 0.05). The test for subgroup effects revealed that treatment duration-related subgroup differences were statistically significant in the CRP (*p* = 0.002, *I*^2^ = 90.1%) but not statistically significant in the overall effective rate (*p* = 0.31, *I*^2^ = 2.4%) and WBC (*p* = 0.19, *I*^2^ = 41.4%) (Supplementary Figure 2).

Subgroup analysis also showed that the integrated medicine treatment significantly improved the rate of fever, fatigue, and cough disappearance on studies of treatment duration < 2 weeks (fever disappearance rate: five trials, RR 1.28, 95% CI 1.11 to 1.47; fatigue disappearance rate: four trials, RR 1.72, 95% CI 1.25 to 2.35; cough disappearance rate: five trials, RR 1.44, 95% CI 1.15 to 1.81). However, the test for subgroup effects revealed that treatment duration-related subgroup differences were not statistically significant (overall effective rate: *p* = 0.31, *I*^2^ = 2.4%; fever disappearance rate: *p* = 0.48, *I*^2^ = 0%; fatigue disappearance rate: *p* = 0.06, *I*^2^ = 70.8%; cough disappearance rate: *p* = 0.05, *I*^2^ = 73.4%) (Supplementary Figure 3).

#### Subgroup Analysis Based on Subtypes of COVID-19

In terms of the fatigue disappearance rate and overall effective rate, there was only one study on the type of severe and there was no difference between the groups. Subgroup analysis showed that the integrated medicine treatment significantly improved the overall effective rate, fatigue disappearance rate, and CRP on non-severe type of COVID-19 (overall effective rate: RR 1.17, 95% CI 1.10 to 1.26; fatigue disappearance rate: RR 1.56, 95% CI 1.18 to 2.07; CRP: WMD −3.53, 95% CI −4.31 to −2.76) (Supplementary Figure 4).

Subgroup analysis also showed that the integrated medicine treatment significantly increased the fever disappearance rate, cough disappearance rate, and WBC regardless of whether the type of COVID-19 is severe or non-severe as compared with the CWM treatment (*p* < 0.05). The test for subgroup effects revealed that COVID-19 type-related subgroup differences were statistically significant in the WBC (*p* = 0.003, *I*^2^ = 88.8%) but not statistically significant in the overall effective rate (*p* = 0.97, *I*^2^ = 0%), fever disappearance rate (*p* = 0.45, *I*^2^ = 0%), fatigue disappearance rate (*p* = 0.65, *I*^2^ = 0%), cough disappearance rate (*p* = 0.89, *I*^2^ = 0%), and CRP (*p* = 0.30, *I*^2^ = 8.7%) (Supplementary Figure 5).

#### Subgroup Analysis Based on Risk Bias for Sequence Generation

Subgroup analysis showed that the integrated medicine treatment significantly improved the overall effective rate, fever disappearance rate, fatigue disappearance rate, cough disappearance rate, CRP level, and WBC count on studies of low risk for sequence generation compared with CWM treatment (*p* < 0.05). However, the test for subgroup effects revealed that risk bias-related subgroup differences were statistically significant in the CRP (*p* = 0.04, *I*^2^ = 75.2%) but not statistically significant in overall effective rate (*p* = 0.45, *I*^2^ = 0%), fever disappearance rate (*p* = 0.12, *I*^2^ = 58.5%), fatigue disappearance rate (*p* = 0.50, *I*^2^ = 0%), cough disappearance rate (*p* = 0.18, *I*^2^ = 44.3%), and WBC (*p* = 0.17, *I*^2^ = 46.5%) (Supplementary Figure 6).

### Evaluation of Publication Bias

Assessment of publication bias using Begg's test showed that there was no potential publication bias among the included trials (fever disappearance rate: *z* = 1.50, *P* = 0.133; cough disappearance rate: *z* = 0.62, *p* = 0.536; CRP: *z* = 0.30, *p* = 0.764; WBC: *z* =0.60, *p* = 0.548).

## Discussion

### Summary of Evidence

Traditional Chinese medicine has a long history and plays an important role in the current medical treatments in China. COVID-19 is a severe viral infection and lacks specific drugs. TCM is involved in the treatment of patients with different degrees of severity in the COVID-19 diagnosis and treatment program of China. Therefore, this study systematically reviewed, summarized, and disseminated the best evidence through strict inclusion and exclusion criteria to provide better evidence for COVID-19 treatment decisions.

In Chinese medicine, the dosage, composition, and course of treatment can be adjusted according to the condition of the patient. After a comprehensive search of seven databases, 19 RCTs included in this meta-analysis used 16 different herbs or proprietary Chinese medicines, which means that, in terms of treatment, Chinese medicine can make more choices for the best treatment compared with Western medicine. Our results showed that clinical symptoms such as fever and fatigue, as well as overall effective rate, chest CT, CRP, and WBC, were more improved in the integrated medicine group than in the CWM group. In addition, our imprecise results also showed that integrated medicine did not improve the cough disappearance rate compared with Western medicine. When we excluded studies that led to increased heterogeneity by sensitivity analysis, we found that the integrated medicine group improved the cough symptoms better than the CWM group. For COVID-19, more than 2 weeks of treatment course is suggested (36). However, our research also shows that, when the treatment time is <2 weeks, the effect of integrated medicine treatment is more obvious in improving overall effective rate, clinical symptoms, CRP level, and WBC count compared with the treatment of CWM. For patients with severe and non-severe COVID-19, integrated medicine is more effective in improving fever and cough symptoms, and WBC count than CWM.

According to the TCM viewpoint, some experts believe that, although, the cold and dampness are blocked in the early stage of COVID-19, the cold and dampness often turn into heat, and it is easy to manifest as damp heat (37, 38). The damp-heat virus invades the lungs from the nose and mouth, causing lung dysfunction and blockage of body fluids. Therefore, patients with COVID-19 usually have a dry cough with little sputum and difficulty breathing. The symptoms of dry cough and lack of sputum are inconsistent with lung pathological changes. During the dissection process, it was found that there was a large amount of mucus secretions and pulmonary interstitial edema in the airways of the patient, but the exudate was very viscous, and these secretions were difficult to discharge (39, 40). Because the terminal airways are blocked by secretions, the patients have severe breathing difficulties. Even if sputum suction, oxygen therapy, and ventilator adjuvant treatment are given, it is not conducive to the removal of deep “phlegm”; instead, it makes the sputum thick or forms sputum scabs, and a large amount of retention in the lungs aggravates lung ventilation dysfunction and even leads to respiratory failure. The changes in chest CT in this study reflect that TCM has a significant effect in improving sputum drainage in the treatment of COVID-19.

Abnormal inflammation indicators are the most common indicators of viral infection. SARS-CoV-2 can also cause immune cascade of the body, resulting in systemic inflammatory response syndrome (SIRS), diffuse intravascular coagulation (DIC), and MODS (41). It has been reported that the stormy release of a large number of inflammatory cytokines correlated with mortality (42). Studies have shown that, in the early stage of COVID-19, CRP levels positively correlate with the lung disease and can reflect the severity of the disease (43). In addition, the baseline levels of CRP can be used as independent predictors of mortality in COVID-19 patients (44). TCM has multiple targets; it not only has antiviral effects, but it also has therapeutic effects in the occurrence, progression, and outcome stages of the cytokine storm. The change in CRP levels in this study reflects the efficacy of TCM in the treatment of COVID-19 to improve inflammation.

In summary, the currently available evidence suggests that integrated medicine treatment can be an effective treatment for COVID-19, when the treatment time is 5–21 days.

### Limitations of the Current Review

Although, we followed the Cochrane method for meta-analysis, conducting comprehensive literature retrieval, repeatedly and independently screening literature, and abstracting data, our meta-analysis still has some limitations.

First, the general information of the patient is not provided in detail, such as the baseline age, underlying disease, subtype of COVID-19 participants, disease course, treatment duration, and the type and dosage of Western medicine used in the control group. Moreover, different laboratory measuring instruments and different normal value ranges may be responsible for the high heterogeneity in laboratory measurement outcomes (CRP, WBC, ESR, PCT, and LY). In addition, the use of different herbs in different interventions may be responsible for the observed high heterogeneity of the pooled effect size estimates. Furthermore, the method of random sequence generation is unclear, and most of the studies lack details of allocation concealment and blinding, leading to possible selection bias and implementation bias in the included studies. The above factors will cause clinical heterogeneity and methodological heterogeneity before performing meta-analyses. At the same time, due to the relatively small number of studies involving ESR (three RCTs), PCT (four RCTs), and LY (four RCTs) in the included studies, the insufficient population sample size may lead to statistical heterogeneity, so we have only described the results of each included RCT.

However, there are many obstacles for the control group drugs to be consistent with the preparations of TCM and to eliminate the unique smell of TCM, which might lead to an unblinding of the study. Therefore, it is difficult to implement double-blind Chinese medicine in clinical trials. In data processing, none of the studies reported cases of withdrawal and loss, and due to the lack of long-term follow-up data, there may be insufficient reports of adverse reactions. Finally, we are unable to assess the effects of integrated medicine on other clinically meaningful endpoints, such as the time when 2019-nCoV RT-PCR is negative, and composite events (the total number of patients diagnosed as type critical and all-cause death).

### Research Implications

Considering the limitations of the current trials, the correct methods of allocation concealment and blinding should be recommended when designing future clinical trials in accordance with the Consolidated Standards of Reporting Trials (CONSORT) (45) and TCM guidelines (Standards for Reporting Interventions in Controlled Trials of TCM) (46). In order to optimize the effectiveness of TCM treatment of COVID-19, the design, quality, and reporting of RCTs should be improved, especially the allocation concealment. Although, blinding may be difficult for patients treated by TCM, blinding should be feasible for medical workers, outcome evaluators, and analysts. In addition, it is necessary to actively explore the preparation of placebos, which may be a way to solve the problem of double-blindness of TCM. Future studies may need to refer to the core outcome sets that have been developed, such as a core outcome set for clinical trials on coronavirus disease 2019 (COS-COVID-2019) (36), as an outcome measure for different subtypes of COVID-19, to avoid wasting research resources. Considering the inaccuracies of the included studies, future RCTs should include larger sample size, longer treatment time, and longer follow-up periods to confirm the efficacy of integrated medicine and to formulate the optimal regimens.

### Clinical Practice Implications

The results of the current meta-analysis suggested that the integrated medicine can improve the symptoms of patients with COVID-19. Even if the treatment time is <2 weeks, compared with only CWM treatment, the effect of integrated medicine in improving symptoms is more obvious. In addition, integrated medicine treatment also can effectively improve the chest CT and infection indicators (CRP and WBC) of patients with COVID-19, which may be related to the promotion of sputum drainage in the lungs and anti-inflammatory by Chinese medicine. However, due to the low quality of the evidence and the small sample size, the results of the meta-analysis of ESR, PCT, and LY need further research and verification. This study provides an initial set of evidence for potentially recommending integrated medicine as a treatment plan for COVID-19. The treatment based on syndrome differentiation is one of the characteristics and advantages of TCM treatment. Therefore, each facility utilizing TCM can choose herbal medicines according to the type of syndrome of COVID-19 when using TCM for treatment or research.

## Data Availability Statement

The original contributions presented in the study are included in the article/Supplementary Material, further inquiries can be directed to the corresponding author/s.

## Author Contributions

BY and Y-MB ran the search strategy. BY, Y-MB, X-ZW, and A-XL collected the data. BY and Y-MB re-checked the data. BY performed the analysis and LS re-checked the analysis. BY and Y-MB assessed the quality of studies and LS re-checked the quality. BY wrote the manuscript. Y-MB, J-ZH, JZ, and JY edited the manuscript. G-JF and LS designed and administrated the study. All the authors have read and approved the manuscript.

## Conflict of Interest

The authors declare that the research was conducted in the absence of any commercial or financial relationships that could be construed as a potential conflict of interest.

## References

[1] Stöhr K Cox N. COVID-19 vaccines: call for global push to maintain efficacy. Nature. (2021) 590:36. 10.1038/d41586-021-00273-y 33531701

[2] Tsang NNY So HC Ng KY Cowling BJ Leung GM Ip DKM. Diagnostic performance of different sampling approaches for SARS-CoV-2 RT-PCR testing: a systematic review and meta-analysis. Lancet Infect Dis. (2021). 10.1016/S1473-3099(21)00146-8 PMC8041361 33857405

[3] Wang D Hu B Hu C Zhu F Liu X Zhang J. Clinical characteristics of 138 hospitalized patients with 2019 novel coronavirus-infected pneumonia in Wuhan, China. JAMA. (2020) 323:1061–9. 10.1001/jama.2020.1585 32031570 PMC7042881

[4] Li Q Guan X Wu P Wang X Zhou L Tong Y. Early transmission dynamics in Wuhan, China, of novel coronavirus-infected pneumonia. N Engl J Med. (2020) 382:1199–207. 10.1056/NEJMoa2001316 31995857 PMC7121484

[5] Singh R Kang A Luo X Jeyanathan M Gillgrass A Afkhami S. COVID-19: current knowledge in clinical features, immunological responses, and vaccine development. FASEB J. (2021) 35:e21409. 10.1096/fj.202002662R 33577115 PMC7898934

[6] Rothlin RP Duarte M Pelorosso FG Nicolosi L Salgado MV Vetulli HM. Angiotensin receptor blockers for COVID-19: pathophysiological and pharmacological considerations about ongoing and future prospective clinical trials. Front Pharmacol. (2021) 12:603736. 10.3389/fphar.2021.603736 33854432 PMC8039444

[7] Yang Y Islam MS Wang J Li Y Chen X. Traditional Chinese medicine in the treatment of patients infected with 2019-new coronavirus (SARS-CoV-2): a review and perspective. Int J Biol Sci. (2020) 16:1708–17. 10.7150/ijbs.45538 32226288 PMC7098036

[8] Ren W Liang P Ma Y Sun Q Pu Q Dong L. Research progress of traditional Chinese medicine against COVID-19. Biomed Pharmacother. (2021) 137:111310. 10.1016/j.biopha.2021.111310 33761591 PMC7857050

[9] Zhang YS Cong WH Zhang JJ Guo FF Li HM. Research progress of intervention of Chinese herbal medicine and its active components on human coronavirus. Zhongguo Zhong Yao Za Zhi. (2020) 45:1263–71. 10.19540/j.cnki.cjcmm.20200219.501 32281335

[10] Luo X Ni X Lin J Zhang Y Wu L Huang D. The add-on effect of Chinese herbal medicine on COVID-19: a systematic review and meta-analysis. Phytomedicine. (2020) 85:153282. 10.1016/j.phymed.2020.153282 32800699 PMC7831541

[11] Sun CY Sun YL Li XM. The role of Chinese medicine in COVID-19 pneumonia: a systematic review and meta-analysis. Am J Emerg Med. (2020) 38:2153–9. 10.1016/j.ajem.2020.06.069 33071103 PMC7342052

[12] Xiong X Wang P Su K Cho WC Xing Y. Chinese herbal medicine for coronavirus disease 2019: a systematic review and meta-analysis. Pharmacol Res. (2020) 160:105056. 10.1016/j.phrs.2020.105056 32622723 PMC7331568

[13] Zhou LP Wang J Xie RH Pakhale S Krewski D Cameron DW. The effects of traditional chinese medicine as an auxiliary treatment for COVID-19: a systematic review and meta-analysis. J Altern Complement Med. (2021) 27:225–37. 10.1089/acm.2020.0310 33252246

[14] Ang L Song E Lee HW Lee MS. Herbal medicine for the treatment of coronavirus disease 2019 (COVID-19): a systematic review and meta-analysis of randomized controlled trials. J Clin Med. (2020) 9:1583. 10.3390/jcm9051583 32456123 PMC7290825

[15] Zhilai Z Jia L Wei Y Yuguang W Lianguo R Ping H. Pilot study on the evaluation standard of the curative effects of traditional Chinese medicine on coronavirus disease 2019 (COVID-19) based on cases analysis. J Tradit Chin Med. (2020) 61:1013–23. 10.13288/j.11-2166/r.2020.12.001

[16] Cumpston M Li T Page MJ Chandler J Welch VA Higgins JP. Updated guidance for trusted systematic reviews: a new edition of the Cochrane handbook for systematic reviews of interventions. Cochrane Database Syst Rev. (2019) 10:Ed000142. 10.1002/14651858.ED000142 31643080 PMC10284251

[17] Hu K Guan WJ Bi Y Zhang W Li L Zhang B. Efficacy and safety of Lianhuaqingwen capsules, a repurposed Chinese herb, in patients with coronavirus disease 2019: a multicenter, prospective, randomized controlled trial. Phytomedicine. (2020) 85:153242. 10.1016/j.phymed.2020.153242 33867046 PMC7229744

[18] Ai X Lin L Xie M Tan X. Effect of integrated traditional 522 Chinese and Western medicine on T lymphocyte subsets of patients with normal type of 523 COVID-19. Guangdong Med J. (2020) 20:746–50. 10.13604/j.cnki.46-5241064/r.2020.08.12

[19] Ding X Zhang Y He D Zhang M Tan Y Yu A. Clinical effect and mechanism of qingfei touxie fuzheng recipe in the treatment of COVID-19. Herald Med. (2020) 39:640–4. 10.3870/j.issn.1004-0781.2020.05.012

[20] Duan C Xia W Zhen C Sun G Li Z Li Q. Clinical observation of jinhua qinggan granules in treating pneumonia infected by novel coronavirus. J Tradit Chinese Med. (2020). 61:1473–77. 10.13288/j.11-2166/r.2020.17.001

[21] Fu X Lin L Tan X. Clinical study on 37 case of COVID- 19 treated with integrated traditional Chinese and Western Medicine. Tradit Chinese Drug Res Clin Pharmacol. (2020) 31:600–4. 10.19378/j.issn.1003-5369783.2020.05.016

[22] Fu X Lin L Tan X. Clinical Observation on Effect of Toujie Quwen 538 Granules in Treatment of COVID-19. Chinese J Exp Tradit Med Formulae. (2020) 26:44–8. 10.13422/j.cnki.syfjx.20201314

[23] He Q Zhang Q Gan X Li X. Clinical efficacy analysis of Buzhong Yiqi Decoction in the treatment of mild new coronavirus pneumonia. J Emerg Tradit Chinese Med. (2021) 30:385–7. 10.3969/j.issn.1004-745X.2021.03.003

[24] Jin W Lu Y Zhao W Tang S Sang X Zhang L. The efficacy of recommended treatments with integrated Chinese and Western medicine on coronavirus disease 2019 (COVID-19) in Sichuan: a clinical trial observation. Pharmacol Clin Chinese Mater Med. (2020) 36:6–10. 10.13412/j.cnki.zyyl.20201110.006

[25] Li Y Zhang W. Evaluation on the clinical effect of traditional Chinese Medicine and Western medicine regimens on COVID-19. Guangming J. Chinese Med. (2020) 35:1273–5.33415866

[26] Liu W Su X Liao X Hu P Mei D Zhang Y. Effect analysis of antiviral drugs combined with traditional Chinese medicine in the treatment of mild new coronavirus pneumonia. Contemp Med Sympos. (2021). 19:159–60. 10.3969/j.issn.2095-7629.2021.02.114556

[27] Qiu M Li Q Zhu D Wang C Sun Q Qian C. Efficacy observation of maxing xuanfei jiedu decoction on moderate COVID- 19 patients. J Emerg Tradit Chinese Med. (2020) 29:1129–30,1132. 10.3969/j.issn.1004-745X.2020.07.001

[28] Sun H Xu F Zhang L Wei C Chen J Wang Q. Study on clinical efficacy of lianhua qingke granule in treatment of mild and ordinary COVID-19. Chinese J Exp Tradit Med Formulae. (2020) 26:29–34. 10.13422/j.cnki.syfjx.20201438564

[29] Wang L Xu M Wang Y Li H Liu N Zuo J. Clinical study on Shengmai Powder combined with Shenling Baizhu Powder in the treatment of common Corona Virus Disease 2019. China J Tradit Chinese Med Pharmacy. (2020) 35:4268–71.

[30] Wang Y Chen L Zhang L Ku B Yu R Zhang X. Clinical efllects of Qingfei Paidu Decoction combined with conventional treatment on patients with coronavirus disease 2019. Chinese Tradit Patent Med. (2021) 43:656–9. 10.3969/j.issn.1001-1528.2021.03.017

[31] Yu P Li Y Wan S Wang Y. Efficacy of Lianhua Qingwen Granules combined with Arbidol in the treatment of mild novel coronavirus pneumonia. Chinese Pharmaceut J. (2020) 55:1042–5. 10.11669/cpj.2020.12.014

[32] Zhang Y Lei L Xu Y Wei D Hu F. Clinical efficacy of jinyinhua oral liquid in the treatment of 80 patients with coronavirus disease 2019. China Pharm. (2020) 29:23–6. 10.3969/j.issn.1006-4931.2020.09.006

[33] Wang JB Wang ZX Jing J Zhao P Dong JH Zhou YF. Exploring an integrative therapy for treating COVID-19: a randomized controlled trial. Chin J Integr Med. (2020) 26:648–55. 10.1007/s11655-020-3426-7 32676976 PMC7364292

[34] Xiao M Tian J Zhou Y Xu X Min X Lv Y. Efficacy of Huoxiang Zhengqi dropping pills and Lianhua Qingwen granules in treatment of COVID-19: a randomized controlled trial. Pharmacol Res. (2020) 161:105126. 10.1016/j.phrs.2020.105126 32781283 PMC7414728

[35] Wu-zhong X Gang W Juan D Wang A. Efficacy of herbal medicine (Xuanfei Baidu decoction) combined with conventional drug in treating COVID-19:A pilot randomized clinical trial. Integr Med Res. (2020) 9:100489. 10.1016/j.imr.2020.100489 32874913 PMC7452296

[36] Jin X Pang B Zhang J Liu Q Yang Z Feng J. Core outcome set for clinical trials on coronavirus disease 2019 (COS-COVID). Engineering. (2020) 6:1147–52. 10.1016/j.eng.2020.03.002 32292626 PMC7102592

[37] Wenliang LV. Interpretation based on the guidelines on prevention and treatment of novel coronavirus pneumonia by Chinese medicine in Hubei Province. World Chin Med. (2020) 15:125–8. 10.3969/j.issn.1673-7202.2020.02.001

[38] Tong X Li X Zhao L Li Q Yang Y Lin Y. Discussion on traditional chinese medicine prevention and treatment strategies of coronavirus disease 2019 (COVID-19) from the perspective of “cold-dampness pestilence”. J Tradit Chin Med. (2020) 61:465–70. 10.13288/j.11-2166/r.2020.06.003

[39] Wang C Xie J Zhao L Fei X Zhang H Tan Y. Alveolar macrophage dysfunction and cytokine storm in the pathogenesis of two severe COVID-19 patients. EBioMedicine. (2020) 57:102833. 10.1016/j.ebiom.2020.102833 32574956 PMC7305897

[40] Fox SE Akmatbekov A Harbert JL Li G Quincy Brown J Vander Heide RS. Pulmonary and cardiac pathology in African American patients with COVID-19: an autopsy series from New Orleans. Lancet Respir Med. (2020) 8:681–6. 10.1016/S2213-2600(20)30243-5 32473124 PMC7255143

[41] Ding YQ Bian XW. Analysis of coronavirus disease-19 (COVID-19) based on SARS autopsy. Zhonghua Bing Li Xue Za Zhi. (2020) 49:291–3. 10.3760/cma.j.cn112151-20200211-00114 32268662

[42] Toldo S Bussani R Nuzzi V Bonaventura A Mauro AG Cannatà A. Inflammasome formation in the lungs of patients with fatal COVID-19. Inflamm Res. (2021) 70:7–10. 10.1007/s00011-020-01413-2 33079210 PMC7572246

[43] Wang L. C-reactive protein levels in the early stage of COVID-19. Med Mal Infect. (2020) 50:332–4. 10.1016/j.medmal.2020.03.007 32243911 PMC7146693

[44] Xu JB Xu C Zhang RB Wu M Pan CK Li XJ. Associations of procalcitonin, C-reaction protein and neutrophil-to-lymphocyte ratio with mortality in hospitalized COVID-19 patients in China. Sci Rep. (2020) 10:15058. 10.1038/s41598-020-72164-7 32929124 PMC7490259

[45] Moher D Hopewell S Schulz KF Montori V Gøtzsche PC Devereaux PJ. CONSORT 2010 explanation and elaboration: updated guidelines for reporting parallel group randomised trials. Int J Surg. (2012) 10:28–55. 10.1016/j.ijsu.2011.10.001 22036893

[46] Cheng CW Wu TX Shang HC Li YP Altman DG Moher D. Consort extension for chinese herbal medicine formulas 2017: recommendations, explanation, and elaboration (Traditional Chinese Version). Ann Intern Med. (2017) 167:W7–20. 10.7326/IsTranslatedFrom_M17-2977_1 28654988

