# Supplementary material for: Efficacy of Integrated Traditional Chinese and Western Medicine for Treating COVID-19: A Systematic Review and Meta-Analysis of RCTs
Source: Front Public Health. 2021 Jul 8;9:622707. doi: 10.3389/fpubh.2021.622707 (PMC8298033; doi:10.3389/fpubh.2021.622707)
Supplement: Supplementary file 1 [file Table_1.DOCX]

**Supplementary Appendix**

**Search Strategy**

**Search strategy PubMed (****Web of Science)**

#1 Search "Coronavirus"[Mesh]

#2 Search (coronavirus* or coronovirus* or coronavirinae* or Coronavirus* or Coronovirus* or "2019-nCoV" or 2019nCoV or nCoV2019 or "nCoV-2019" or "COVID-19" or COVID19 or "CORVID-19" or CORVID19 or "WN-CoV" or WNCoV or "HCoV-19" or HCoV19 or CoV or "2019 novel*" or Ncov or "n-cov" or "SARS-CoV-2" or "SARSCoV-2" or "SARSCoV2" or "SARS- CoV2" or SARSCov19 or "SARS-Cov19" or "SARSCov-19" or "SARS-Cov-19" or Ncovor or Ncorona* or Ncorono* or NcovWuhan* or NcovHubei* or NcovChina* or NcovChinese*) Field: Title/Abstract

#3 Search ((#1) OR #2)

#4 Search (“Chinese herbal medicine” or “Traditional Chinese medicine” or “classical Chinese herbal formulas” or “classical Chinese herbal formulas” or “Chinese herb or herbal Medicine” or “herb therapy” or “herbal remedy”) Field: Title/Abstract

#5 Search (#3) AND #4

**Search strategy Embase**

1 coronavirus.mp. or Coronavirinae/

2 exp Coronavirinae/

3 (coronavirus* or coronovirus* or coronavirinae* or Coronavirus* or Coronovirus* or Wuhan* or Hubei* or Huanan or "2019-nCoV" or 2019nCoV or nCoV2019 or "nCoV-2019" or "COVID-19" or COVID19 or "CORVID-19" or CORVID19 or "WN-CoV" or WNCoV or "HCoV-19" or HCoV19 or CoV or "2019 novel*" or Ncov or "n-cov" or "SARS-CoV-2" or "SARSCoV-2" or "SARSCoV2" or "SARS-CoV2" or SARSCov19 or "SARS-Cov19" or "SARSCov-19" or "SARS-Cov-19" or Ncovor or Ncorona* or Ncorono* or NcovWuhan* or NcovHubei* or NcovChina* or NcovChinese*).mp.

4 1 or 2 or 3

5 (“Chinese herbal medicine” or “Traditional Chinese medicine” or “classical Chinese herbal formulas” or “classical Chinese herbal formulas” or “Chinese herb or herbal Medicine” or “herb therapy” or “herbal remedy”).mp.

6 4 and 5

**Figure**


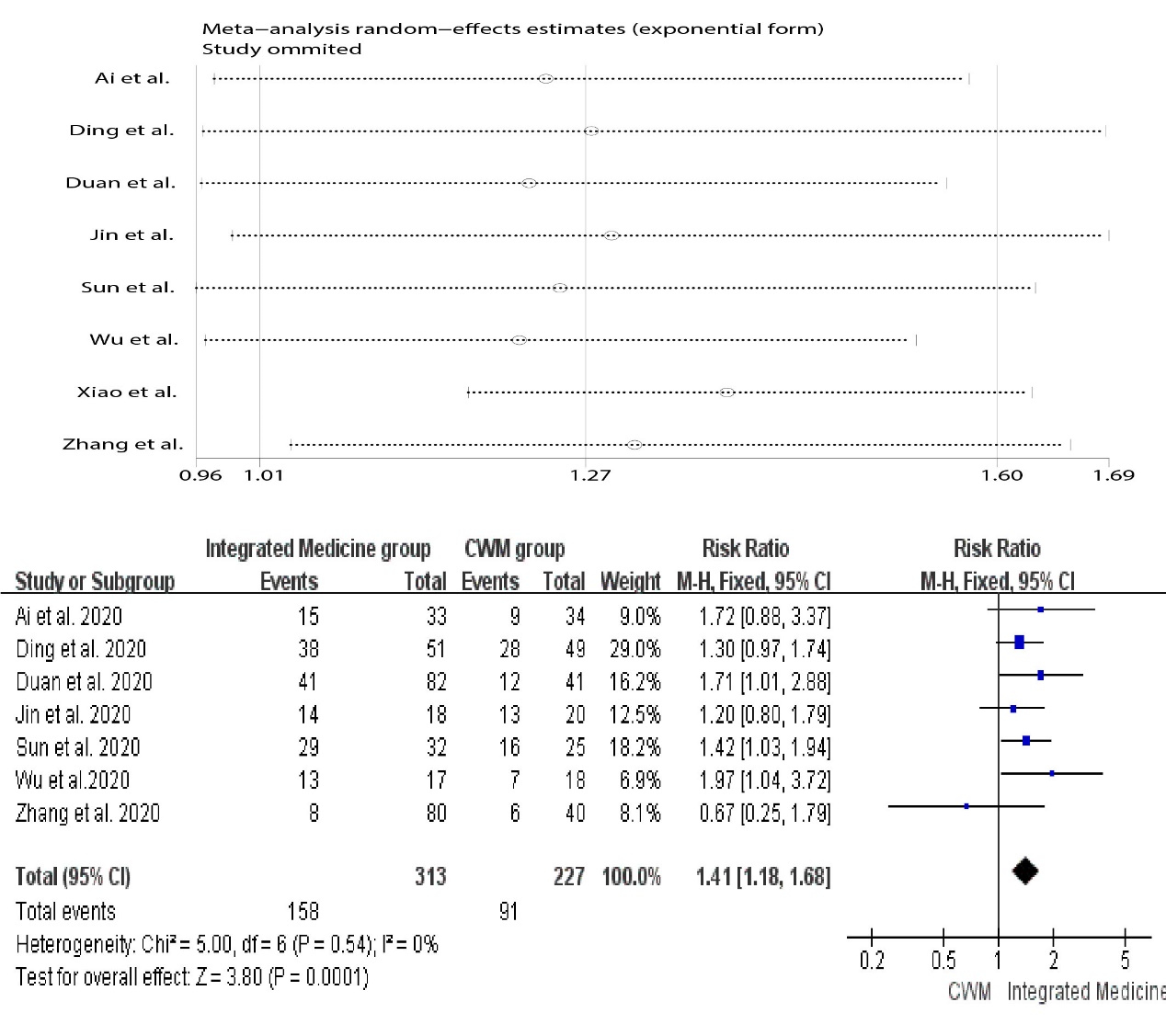


**FIGURE S1 Sensitivity analysis plots of cough disappearance rate**


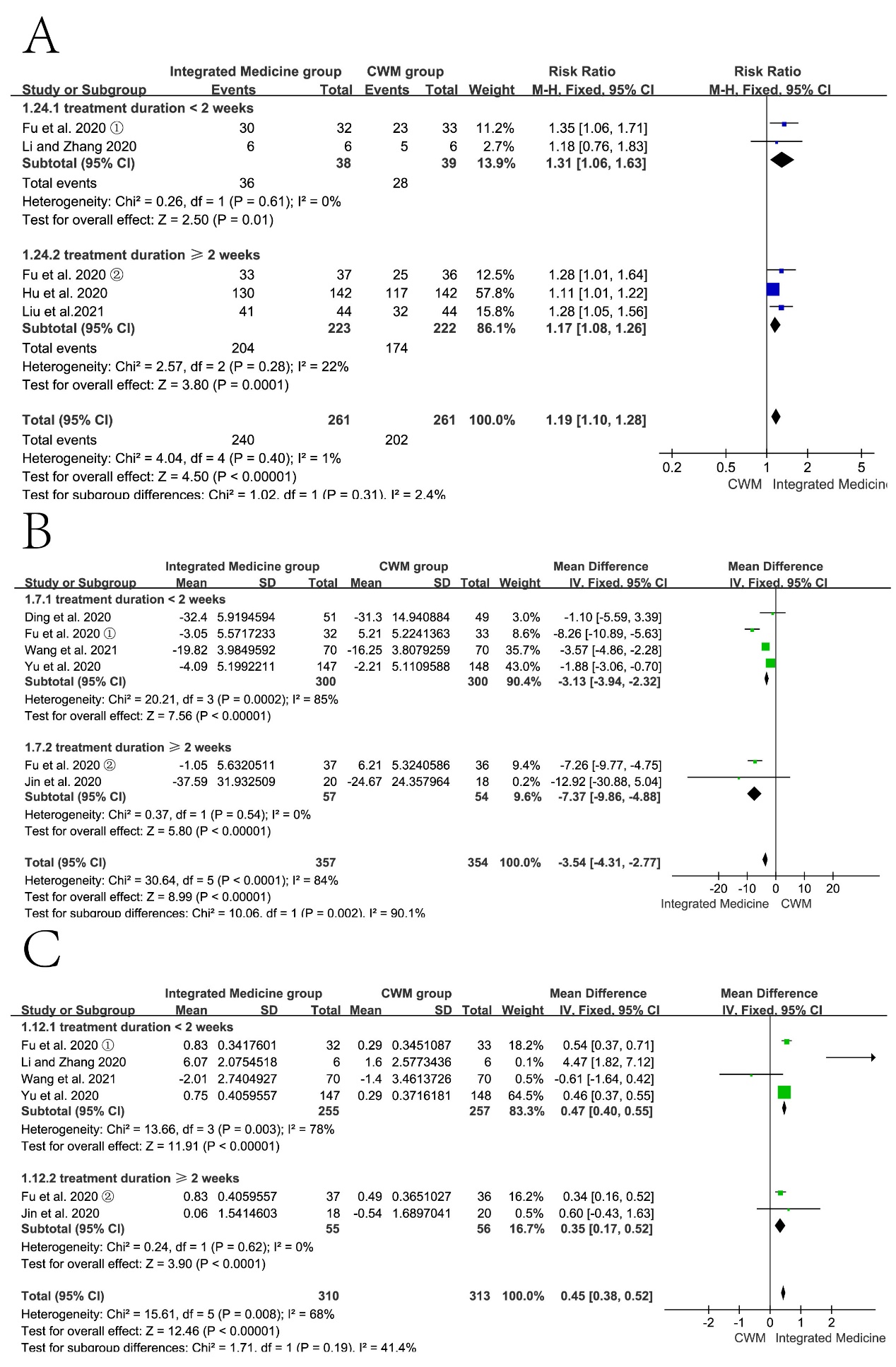


**FIGURE S2** Forest plot comparing the overall effective rate (A), CRP (B) and WBC (C) for Integrated Medicine versus CWM based on treatment duration

**
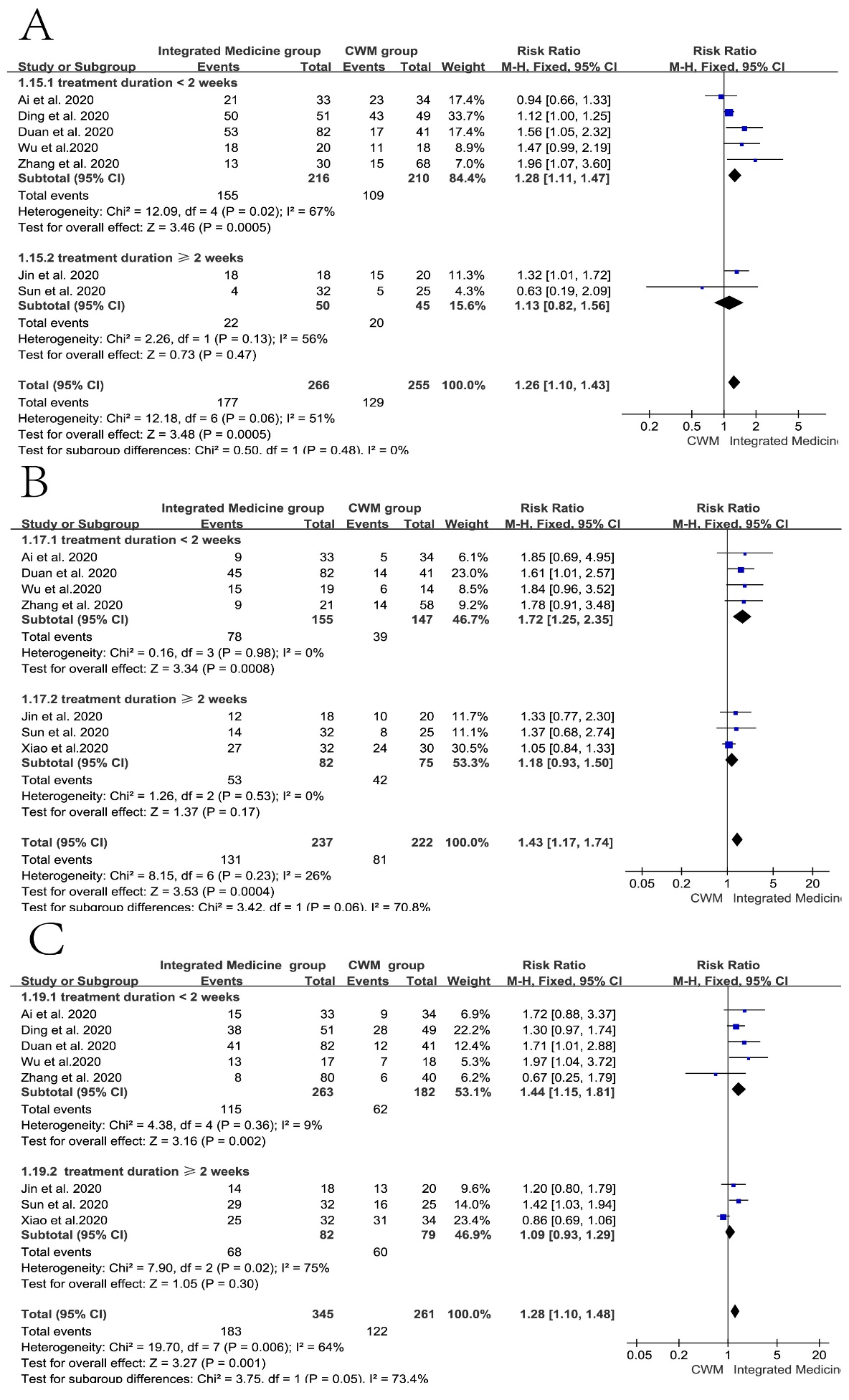
**

**FIGURE S3** Forest plot comparing the fever disappearance rate (A), fatigue disappearance rate (B) and cough disappearance rate (C) for Integrated Medicine versus CWM based on treatment duration


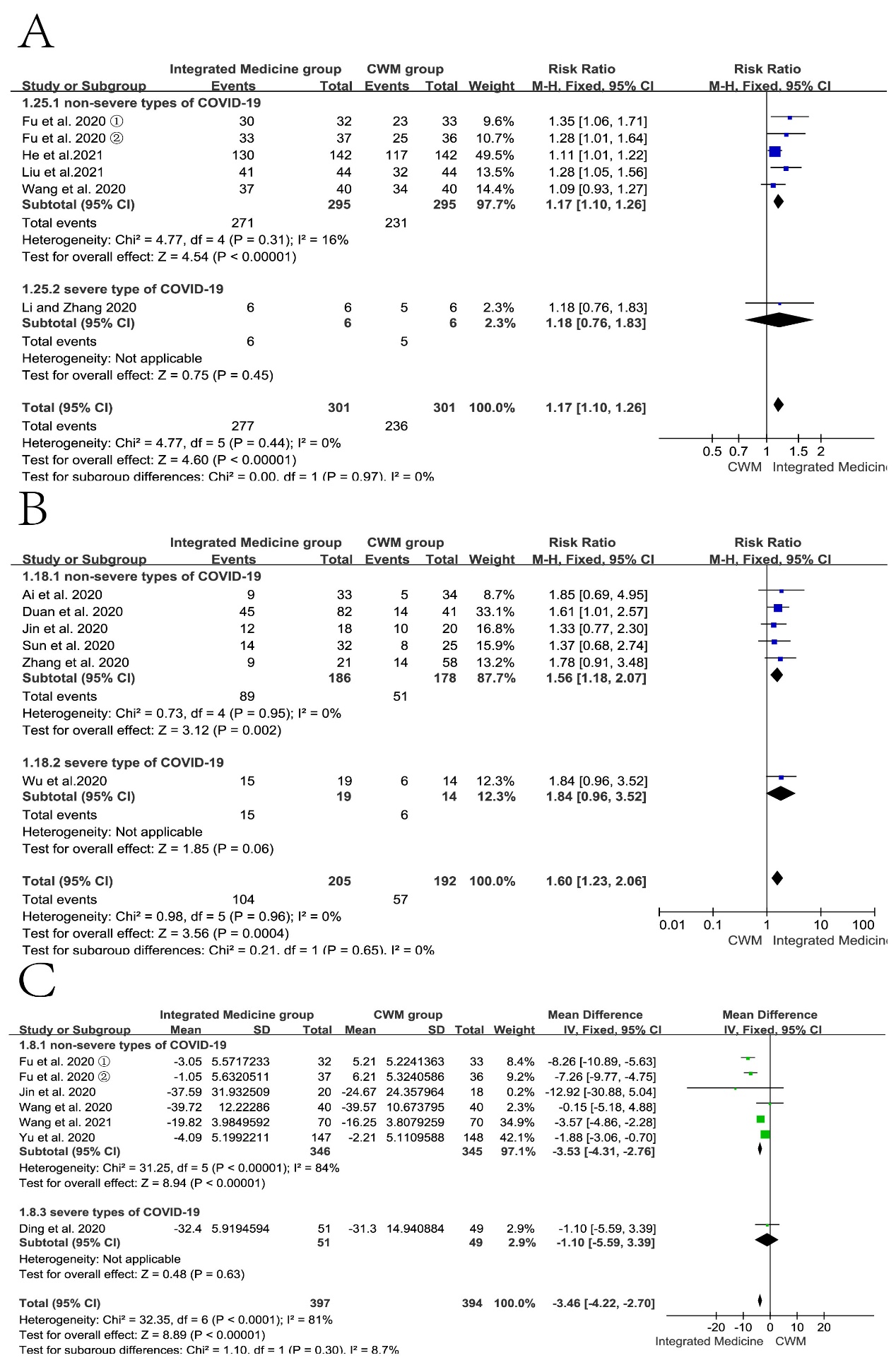


**FIGURE S4** Forest plot comparing the overall effective rate (A), fatigue disappearance rate (B) and CRP (C) for Integrated Medicine versus CWM based on subtypes of COVID-19


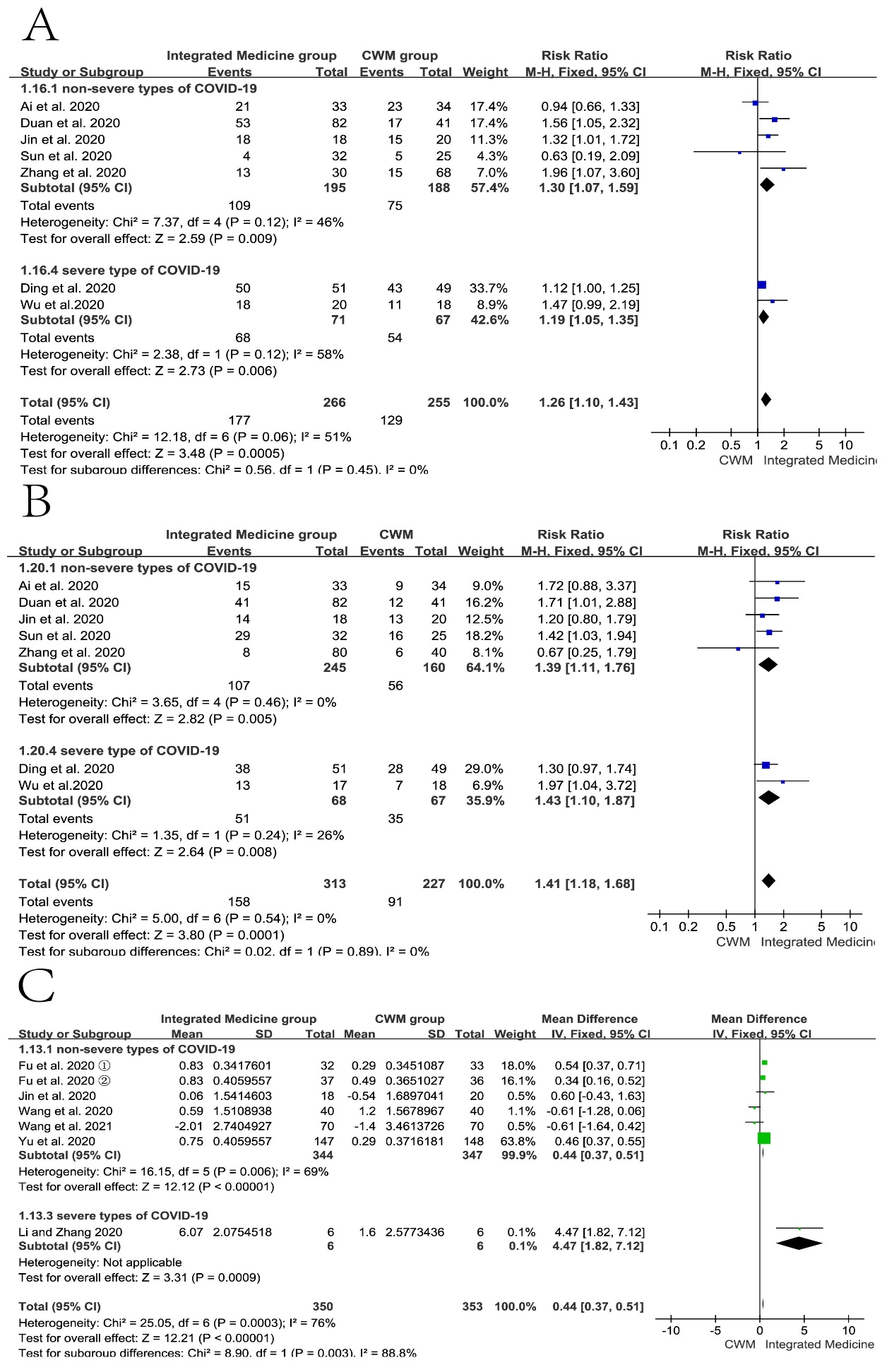


**FIGURE S5** Forest plot comparing the rate of fever disappearance (A), cough disappearance (B) and WBC (C) for Integrated Medicine versus CWM based on subtypes of COVID-19


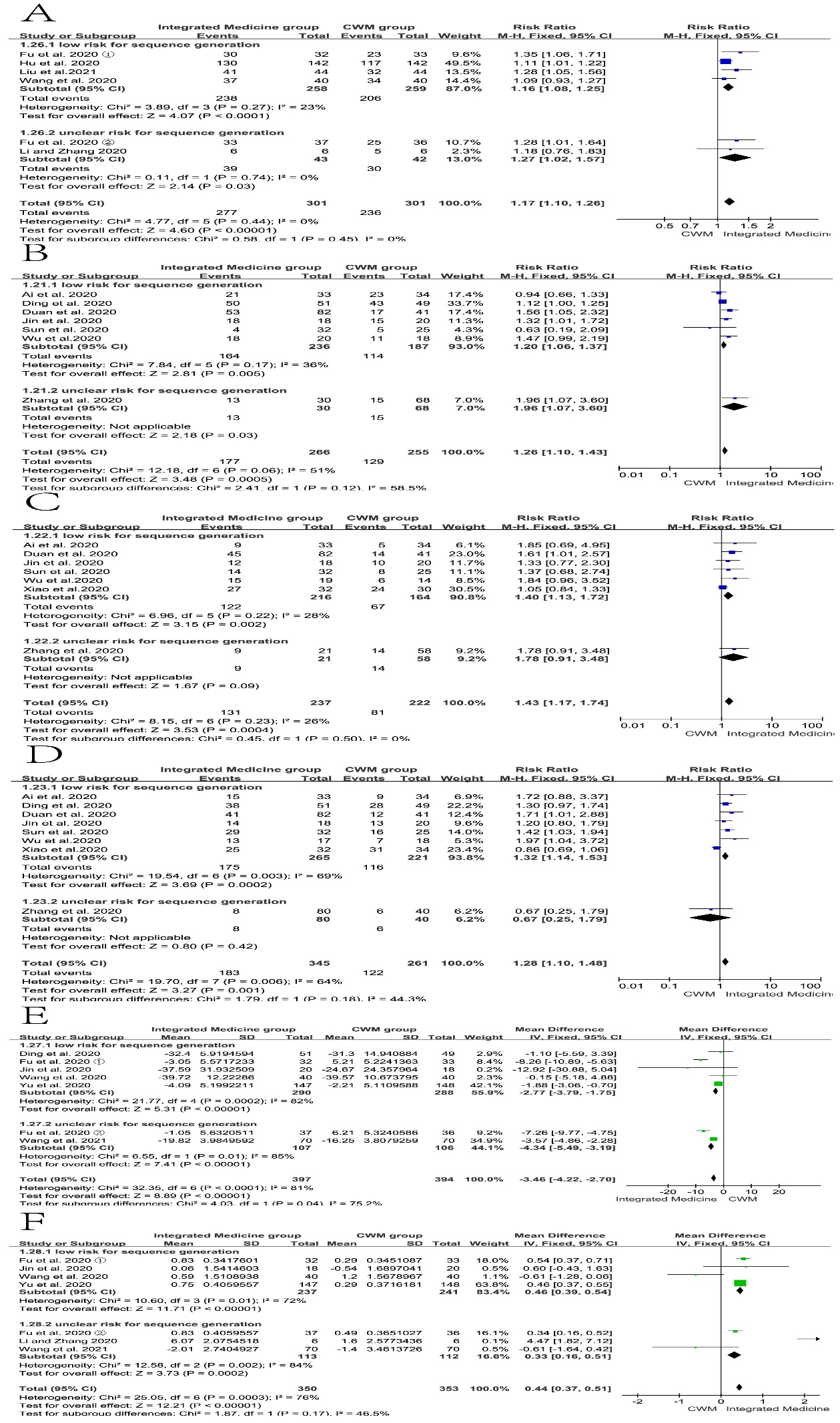


**FIGURE S6** Forest plot comparing the overall effective rate (A), fever disappearance rate (B), fatigue disappearance rate (C), cough disappearance rate (D), CRP (E), and WBC (F) for Integrated Medicine versus CWM based on risk bias for sequence generation
